# Downregulation of Inflammatory Cytokine Release from IL-1β and LPS-Stimulated PBMC Orchestrated by ST2825, a MyD88 Dimerisation Inhibitor

**DOI:** 10.3390/molecules25184322

**Published:** 2020-09-21

**Authors:** Sergio Ramírez-Pérez, Luis Alexis Hernández-Palma, Edith Oregon-Romero, Brian Uriel Anaya-Macías, Samuel García-Arellano, Guillermo González-Estevez, José Francisco Muñoz-Valle

**Affiliations:** 1 Instituto de Investigación en Ciencias Biomédicas (IICB), CUCS, Universidad de Guadalajara, Guadalajara, Jalisco 44340, Mexico; ser.ramirez_iq@hotmail.com (S.R.-P.); alexishdz@outlook.es (L.A.H.-P.); oregon_edith@hotmail.com (E.O.-R.); briananayamacias@gmail.com (B.U.A.-M.); sm_0490@hotmail.com (S.G.-A.); guillermo.gonzalezestevez@cucs.udg.mx (G.G.-E.); 2 Department of Orthopaedics, School of Medicine, Emory University, Atlanta, GA 30322, USA; 3 Instituto Transdisciplinario de Investigación y Servicios (ITRANS), Universidad de Guadalajara, Zapopan, Jalisco 45150, Mexico

**Keywords:** ST2825, MyD88 inhibition, cytokine downregulation, PBMC

## Abstract

The inflammatory process implicates homeostasis disruption and increased production of inflammatory mediators. Myeloid differentiation primary response 88 (MyD88) is an essential protein recruited after lipopolysaccharide (LPS) and interleukin (IL)-1β stimulation, a process that converges in nuclear factor kappa B (NF-κB) activation, as well as a transcription of several genes of both pro- and anti-inflammatory cytokines. The inhibition of MyD88 has shown efficacy by decrease inflammatory response, and has demonstrated potential application as a therapeutic target in chronic diseases. In this study, we investigate the effect of MyD88 dimerisation inhibitor ST2825 on cytokine production from rhIL-1β and LPS-stimulated peripheral blood mononuclear cells (PBMC) from healthy blood donors (HBD). ST2825 significantly downregulates the production of IFN-γ, IL-6, IL-12, IL-2, IL-15, IL-7, VEGF, IL-1Ra, IL-4, IL-5, IL-13 and IL-9 (*p* < 0.05) in LPS-stimulated PBMC. Moreover, ST2825 had a relatively low impact on IL-1β signalling pathway inhibition, showing that only a few specific cytokines, such as IFN-γ and IL-1Ra, are inhibited in rhIL-1β-stimulated PBMC (*p* < 0.01). In conclusion, MyD88 dimerisation inhibitor ST2825 showed high efficacy by inhibiting pro- and anti-inflammatory cytokine production in LPS-stimulated PBMC. Moreover, although rhIL-1β induced a sustained cytokine production (*p* < 0.05), ST2825 did not show a significant effect in the secretion of neither pro- nor anti-inflammatory cytokines in rhIL-1β-stimulated PBMC.

## 1. Introduction

Myeloid differentiation primary response 88 (MyD88) represents an important molecule associated with activation of several signalling pathways, which are implicated in the inflammatory immune response [1]. Interleukin (IL)-1β and Toll-like receptors (TLR) signalling pathways are one of the most strongly studied mechanisms in which MyD88 plays a primary role [2,3,4,5].

The IL-1β activity implicates binding between this cytokine and the IL-1R type I (IL-1RI), this binding triggers the interaction of Toll-IL-1-receptor (TIR) domains followed by MyD88 recruitment and downstream signalling cascades, which converge in the activation of several transcription factors and pro-inflammatory cytokine production [2,6]. Regarding inflammation-associated TLR activation, TLR4 has been the best-studied molecule in both innate and adaptive immunity [7,8]. TLR4 signalling pathway can be activated MyD88-dependent or independent manner [7]; nevertheless, TLR4-dependent LPS-regulated signalling involves the recruitment and homodimerisation of MyD88 and leads to pathways of intracellular signal transduction which converge in the production of inflammatory mediators implicated in the regulation of the inflammatory process [8,9]. 

Overactivated MyD88-dependent LPS and IL-1β inflammatory signalling pathways have displayed high expression of pro-inflammatory mediators not only in healthy blood donors (HBD), but also in patients with chronic, systemic and autoimmune diseases [10,11,12,13,14,15]. Due to this fact, the identification of new molecules that regulate these signalling pathways has taken high relevance [5,11,16,17,18]. 

The chemical molecule ST2825 acts as an inhibitor of MyD88 dimerisation and its activity has been demonstrated through the inhibition of TLR9-dependent CpG-regulated signalling, and inhibition of IL-12, IL-1β, IL-6 and tumor necrosis factor alpha (TNF-α) expression in LPS-stimulated RAW 264.7 cells [19,20,21,22]. However, the effect of the ST2825 molecule on cytokine production mediated by IL-1β and LPS-stimulated peripheral blood mononuclear cells (PBMC) has yet been clarified.

In the present study, PBMC obtained from HBD were stimulated with both rhIL-1β and LPS to identify pro- and anti-inflammatory cytokine profiles, as well as, the inhibitory activity of ST2825 molecule on the cytokine secretion. Our results indicate that inhibition of MyD88 dimerisation mediated by ST2825 molecule causes a decrease secretion of both pro- and anti-inflammatory cytokines in supernatants of LPS-stimulated PBMC; however, ST2825 showed high impact neither pro- nor anti-inflammatory cytokine secretion in rhIL-1β-stimulated PBMC.

## 2. Results 

### 2.1. Inhibitory Activity of ST2825 Molecule in rhIL-1β and LPS-Stimulated PBMC

**Figure 1:**
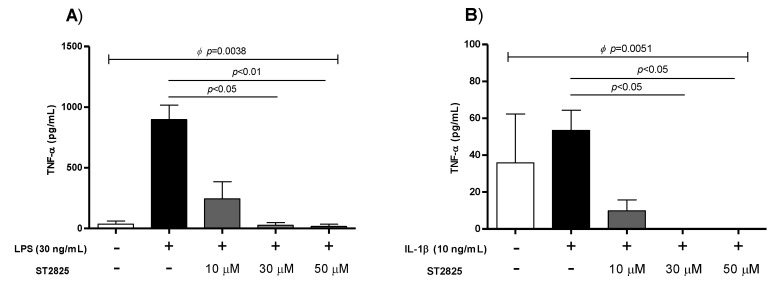
Inhibition curve for LPS and rhIL-1β mediated by ST2825 molecule. (**A**) The soluble levels of TNF-α in the supernatant of LPS-stimulated peripheral blood mononuclear cells (PBMC) at 30 ng/mL and LPS (30 ng/mL) plus different concentrations of ST2825 (10, 30 and 50 μM) were determined. (**B**) The soluble levels of TNF-α in the supernatant of rhIL-1β-stimulated PBMC at 10 ng/mL and rhIL-1β (10 ng/mL) plus different concentrations of ST2825 (10, 30 and 50 μM) were determined. Significant inhibition was identified at 30 μM (*p* < 0.05) and 50 μM (*p* < 0.01) of ST2825 for LPS; while for rhIL-1β significant inhibition was identified at 30 μM (*p* < 0.05) and 50 μM (*p* < 0.05) of ST2825. Data provided in medians and interquartile ranges (n = 4), *^Φ^* Kruskal-Wallis test was performed, and Dunn’s test obtained statistically significant differences.

To determine the specific concentration of ST2825 in which the secretion of cytokines was inhibited, curves of different concentrations of ST2825 were performed (Figure 1). TNF-α quantification was taken as a positive control, and the concentration of this cytokine was determined in supernatants of LPS-stimulated PBMC after 24 h (Figure 1A). The concentration of TNF-α in supernatants of PBMC without stimuli was 36.62 pg/mL; whereas, a high concentration of TNF-α in LPS-stimulated PBMC was identified (906.7 pg/mL). Regarding ST2825 stimuli three different concentrations were taken 10, 30 and 50 μM and TNF-α levels were determined, the medium values were 234.6 pg/mL (*p* = not significant [n.s.]), 28.33 pg/mL (*p* < 0.05), and 15.60 pg/mL (*p* < 0.01), respectively. Similarly, the concentration of TNF-α was determined in supernatants of rhIL-1β-stimulated PBMC (Figure 1B). TNF-α levels from rhIL-1β-stimulated PBMC were 51.78 pg/mL, for rhIL-1β plus 10 μM of ST2825 were 8.24 pg/mL (*p* = n.s.), and after add 30 and 50 μM of ST2528 to rhIL-1β-stimulated PBMC, the TNF-α levels were 0 pg/mL for both (*p* < 0.05) (Figure 1B). 

### 2.2. Inhibition of Pro-Inflammatory Cytokines Orchestrated by ST2825 in LPS-Stimulated PBMC 

**Figure 2:**
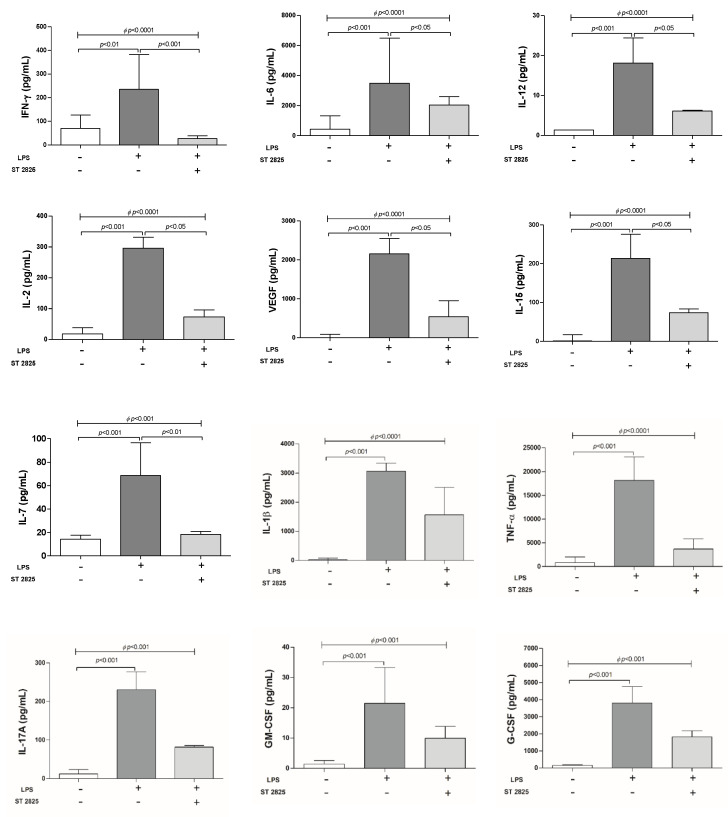
Effect of ST2825 on inhibition of pro-inflammatory cytokine secretion in the supernatant of LPS-stimulated PBMC. The soluble pro-inflammatory cytokines were determined in the PBMC supernatant of HBD (n = 10). PBMC (1 × 10^6^ cells per well at a final volume of 1 mL) were cultured for 24 h under three different conditions: First, RPMI medium alone; second, LPS (30 ng/mL) and third, LPS (30 ng/mL) plus ST2825 (30 μM). Data provided in median and interquartile ranges (*^Φ^* Kruskal-Wallis test and multiple comparisons by Dunn’s test).

LPS has been implicated in the production of pro-inflammatory cytokines through TLR4 activation. Our results indicate that LPS is a potent inductor of several pro-inflammatory cytokines in PBMC. Statistically significant differences were found between PBMC treated with RPMI alone and LPS (*p* < 0.01). In addition, ST2825 molecule was used as a negative regulator of TLR4-dependent LPS-regulated signalling pathway. ST2825 in LPS-stimulated PBMC decreased secretion of interferon gamma (IFN-γ) (*p* < 0.001), IL-6 (*p* < 0.05), IL-12 (*p* < 0.05), IL-2 (*p* < 0.05), vascular endothelial growth factor (VEGF) (*p* < 0.05), IL-15 (*p* < 0.05) and IL-7 (*p* < 0.01) (Figure 2; Table S1). Since our study included males and females; in order to identify differential effects on cytokine production release a statistical analysis by gender was performed. However, our results showed non-statistically significant differences between males and females (data not shown). 

As a control for the effect of ST2825 alone, a statistical analysis was performed by comparing the production of inflammatory cytokines studied in the presence or absence of ST2825. For IFNγ, TNFα, IL-1Ra and IL-2, statistically significant differences were found (*p* < 0.05). The levels of these cytokines in the presence of ST2825 were significantly lower than in PBMC treated with RPMI alone; furthermore, a higher cytokine secretion as an effect of ST2825 than in the basal response of untreated PBMC was not observed (Table S2). 

### 2.3. Inhibition of Anti-Inflammatory Cytokines Orchestrated by ST2825 in LPS-Stimulated PBMC 

**Figure 3:**
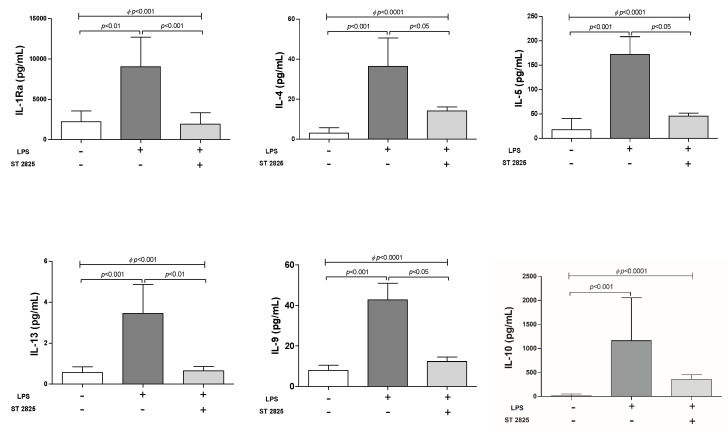
Effect of ST2825 on inhibition of anti-inflammatory cytokine secretion in the supernatant of LPS-stimulated PBMC. The soluble pro-inflammatory cytokines were determined in the PBMC supernatant of HBD (n = 10). PBMC (1 × 10^6^ cells per well at a final volume of 1 mL) were cultured for 24 h under three different conditions: First, RMPI medium alone; second, LPS (30 ng/mL) and third, LPS (30 ng/mL) plus ST2825 (30 μM). Data provided in median and interquartile ranges (*^Φ^* Kruskal-Wallis test and multiple comparisons by Dunn’s test).

The concentration of anti-inflammatory cytokines was determined in supernatants of LPS-stimulated PBMC. Interestingly and contrary to expectations, after 24 h of stimulation with LPS in PBMC, we observed anti-inflammatory cytokine production of IL-1Ra, IL-4, IL-5, IL-13, IL-10 and IL-9 (*p* < 0.01). Additionally, ST2825 inhibited the secretion of IL-1Ra (*p* < 0.001), IL-4 (*p* < 0.05), IL-5 (*p* < 0.05), IL-13 (*p* < 0.01) and IL-9 (*p* < 0.001), but not IL-10 (354.7 pg/mL, *p* = n.s.) (Figure 3; Table S1). Moreover, it was observed that anti-inflammatory cytokine secretion from PBMC stimulated with ST2825 alone, have a similar response to unstimulated cells (Table S2). Based on these results, PBMC stimulated with LPS can secrete pro- and anti-inflammatory cytokines and ST2825 can inhibit the observed response; this is a relevant finding that has not been reported till date.

### 2.4. Inhibition of Pro- and Anti-Inflammatory Cytokines Orchestrated by ST2825 in rhIL-1β-Stimulated PBMC 

The role of IL-1β has been widely described in several chronic conditions, as well as in several cell types. Our results showed high secretion of pro-inflammatory cytokines in rhIL-1β-stimulated PBMC (Table 1). In an exciting way and as previously reported, IL-1β represents an important cytokine capable of inducing Th17-related cytokine profile. In this study, we observed high production of these cytokines: However, only IL-17A, granulocyte-monocyte colony-stimulating factor (GM-CSF) and granulocyte colony-stimulating factor (G-CSF) increased significantly after 24 h of rhIL-1β stimulation (*p* < 0.05). Furthermore, the concentration of cytokines, such as IL-12 (*p* < 0.01), VEGF (*p* < 0.05) and IL-15 (*p* < 0.05), were found higher after rhIL-1β stimulation. On the other hand, only IL-4 and IL-9 significantly increased after rhIL-1β stimulation (*p* < 0.01). 

Regarding the ST2825 effect on rhIL-1β-stimulated PBMC and contrary to our expectations; our results showed that this molecule had a relatively low impact on the IL-1β signalling pathway inhibition (Table 1). ST2825 molecule only inhibited the secretion of IFN-γ (*p* < 0.01) and IL-1Ra (*p* < 0.001). The present study shows that the specific inhibition of critical components in the IL-1 signalling pathway is not enough to avoid the secretion of inflammatory mediators, the above suggests that various MyD88-independent mechanisms could regulate the production of cytokines in PBMC.

## 3. Discussion 

The role of inflammatory key mediators, such as IL-1β and LPS, in activating the innate immune response and subsequently leading to inflammation has been widely described in several studies [2,3]. IL-1β and LPS-signalling pathways trigger cascades of intracellular activation mediated by MyD88 recruitment, which converges in the activation of transcription factors, such as NF-κB and its translocation to the nucleus [2,3,23,24]. The expression of receptors associated with these pro-inflammatory mediators can be given in a variety of cells of the immune system, within the main ones are professional antigen-presenting cells, Treg cells and effector T cells [24,25,26,27].

Our results show that after 24 h, pro-inflammatory cytokine levels of IL-1β, TNF-α, IFN-γ, IL-6, IL-12, IL-17A, G-CSF, GM-CSF, IL-2, VEGF, IL-15 and IL-7 significantly increased in LPS-stimulated PBMC. Recent studies performed in vitro have described that in both PBMC and THP-1 cells can be possible to carry out a differentiation towards M1 macrophages by stimulation with LPS, IFN-γ or GM-CSF; M1 macrophages have been characterised by the expression of pro-inflammatory cytokines, such as IL-6, IL-12, TNF-α and IL-1β [13,28,29]. Regarding cytokines, such as IL-6 and TNF-α, previous studies performed in LPS-stimulated PBMC from HBD reported increased levels of these cytokines in comparison with those levels found in unstimulated PBMC [13,30]. An important cytokine involved in the innate immune response mediated by NK and NKT cells is IL-15, which increased after LPS stimulation; concerning this result, one study reported that LPS-stimulated monocytes could produce IL-15 [9]. Concerning the expression of GM-CSF and VEGF, previous studies conducted in LPS-stimulated PBMC after 24 h showed high levels of both cytokines, as we observed in this study [15]. An earlier report indicated that GM-CSF expression increased in LPS-stimulated PBMC; nonetheless, its expression remains low compared with other pro-inflammatory cytokines, such as TNF-α or IL-6, which could suggest that this cytokine is produced only for a specific population of monocytes [13].

The activation of several transcription factors after LPS stimulation has been demonstrated in a large number of studies. Rothfuchs et al. reported that IFN-γ mRNA expression was strongly diminished in TLR4^-/-^ macrophages compared with the wild type phenotype; moreover, TLR4 activation causes MyD88-dependent IFN-α production, which results in an autocrine effect that regulates IFN-γ mRNA expression by STAT1 activation [31]. On the other hand, increased expression of ROR-γt and the phosphorylated form of NF-κB1 were found after LPS stimulation, and this effect was associated with the differentiation of Th17 cells and high expression of IL-17A [32]. Peyssonnaux et al. reported that HIF-1α was highly expressed in LPS-stimulated macrophages; interestingly, HIF-1α/RORγt/p300 complex can be linked to the promoter region of IL17A and can also promote its transcription [9,33,34]. VEGF is a cytokine produced in response to the hypoxia process and subsequently to HIF-1α activation which, as mentioned above, HIF-1α is a transcription factor induced in response to LPS [15,33].

Transcription factor activation followed by high production of pro-inflammatory cytokines in a TLR4-LPS-dependent pathway has been widely described. However, posttranscriptional mechanisms implicated in TLR4 activation have also recently been described. For example, Arid5a protein is a crucial factor for the production of IL-6; this protein binds to the 3’-UTR region of the IL-6 mRNA and leads to its stabilisation and subsequently, to its efficient expression in vivo [35]. Moreover, Nyati et al. described an alternative LPS signalling pathway that is independent on p38 activation, but dependent on the mitogen-activated protein kinase (MAPK) phosphatase 1 (MKP-1) [35]. This phosphatase MKP-1 can induce the translocation from the nucleus to the cytoplasm of AU-rich element RNA-binding protein 1 (AUF-1), and AUF-1 acts by stabilising the mRNA of IL-6, TNF-α and IL-10 [35].

According to the present study, we observed that the production of anti-inflammatory cytokines significantly increases in LPS-stimulated PBMC. Several studies have reported that TLR4-dependent LPS-regulated signalling pathway causes a predominantly pro-inflammatory response in PBMC. However, some interesting studies have reported that the expression of IRF4 could also be expressed after LPS/IFN-γ or IL-4 stimulation; IRF4 is a key transcription factor involved in the differentiation of M2 macrophages [28,36,37]. However, a significant limitation in our study is that the expression of cytokines was determined after 24 h of stimulation. In this regard, several reports indicate that pro-inflammatory cytokines have an early maximum expression peak within the first eight hours of LPS stimulation (IL-12, IL-1β, IL-6, TNF-α and IL-15) and expression of cytokines, such as IL-10 and IL-1Ra, exhibit a maximum expression peak at 20 and 48 h, respectively [9]. This behaviour on the cytokine profile could also be explained by a process known as “TLR tolerance”, which is characterised by reducing expression of pro-inflammatory cytokines and high expression of M2 activation markers after sustained exposure to TLR ligands [38]. Additionally, a specific type of M2 polarisation in human mononuclear cells after induction of LPS tolerance has been reported [38], and after LPS tolerance recovering, macrophages can express both M1 and M2 polarisation states [39]. 

Stimulation with rhIL-1β triggered the production of several pro-inflammatory cytokines. Interestingly, as previously described in other studies, this cytokine induces the release of Th17-related cytokine profile; IL-17A, GM-CSF and G-CSF were significantly expressed in our research [40,41,42]. Nevertheless, a new T helper cell subset characterised by high production of GM-CSF has been currently described [43]. This cell subset was identified as GM-CSF producing CD4^+^ T cell (TH-GM-CSF), and its differentiation depends on IL-1β signalling pathway, and subsequent IRAK1 and NF-κB activation [43]. Furthermore, TH-GM-CSF cells are able to produce high concentrations of pro-inflammatory cytokines, such as IL-12, IL-6 and TNF-α [43]. Our results also showed increased expression of IL-12 and VEGF; regarding IL-12 production, Langlet et al. reported that this cytokine could be expressed in an IL-1β-dependent activation [44]. Moreover, previous reports have shown that VEGF expression requires activation of HIF-1α, and activation of HIF-1α occurs after IL-1β stimulation [45,46,47]. 

Concerning the results of the inhibition of MyD88 dimerisation and according to expectations, ST2825 molecule significantly inhibits the pro-inflammatory and anti-inflammatory cytokine production mediated by the activation of LPS-TLR4 pathway. About these results, Long et al. reported a significant decrease in cytokine expression of IL-12, IL-1β, IL-6 and TNF-α from RAW264.7 cells treated with LPS plus ST2825 [22]. Several studies have reported that ST2825 causes decreased recruitment and activation of specific molecules and transcription factors involved in the activation of TLR4 and the LPS-dependent immune response activation. The main inhibited molecules, due to the activity of ST2825, are IRAK1, IRAK4, TRAF6, p-IKK, p-IkBα, p-NF-κB and HIF-1α [48,49]. ST2825 has also been proposed as a novel drug targeting in diseases like lymphoma, leukaemia, human hepatocellular carcinoma, and traumatic brain injury [50,51,52]. However, its role as a possible inhibitor in the production of cytokines produced after stimulation with LPS remains undetermined. The decrease recruitment of molecules implicated in the Myddosome formation may explain the inhibition of pro- and anti-inflammatory cytokines as a consequence of TLR4-dependent LPS-regulated signalling.

At the same time, signalling pathway activation after IL-1-IL-1RI-IL-1RAcP complex formation has been widely described [3,6,23,53,54]. This process implicates recruitment and homodimerisation of MyD88, as well as intracellular signalling cascades that converge in the transcription of genes of pro-inflammatory mediators [3,6,23,53,54]. Specific MyD88 dimerisation inhibition has been tested in many studies where the role of this molecule in an IL-1β-dependent activation pathway was evident [21,55,56]. Another study performed in healthy human articular chondrocytes reported decreased MAP kinase (MAPK) activation after MyD88 dimerisation inhibition and IL-1β stimulation [57]. An interesting study conducted by Wang et al. (2019) previously reported that ST2825 decreases the expression of several molecules involved in the Myddosome formation, such as phosphorylated BTK and IκB, along with the decreased secretion of IL-10 and IFN-β from B-cell lymphoma cell lines [58]. In our study, IL-10 release decreases on both IL-1β and LPS-stimulated PBMC treated with ST2825; nonetheless, the effect was not statistically significant. In relation to the IFN response, a decreased secretion of IFN-γ on both IL-1β and LPS-stimulated PBMC was observed our study as an effect of ST2825.

However, our results did not show a substantial inhibitory effect on cytokine production from PBMC treated with IL-1β plus ST2825. In regard to this result, Loiarro et al. (2007) previously described an inhibitory effect observed on NF-κB activity after stimulation with IL-1β stimulation (30 ng/mL) plus ST2825 (30 µM) on HEK293T cells [19]. Nevertheless, although NF-κB activity decreases, ST2825 did not deplete NF-κB activation, which could provide signalling activation enough to produce some of the inflammatory cytokines observed in our study [19]. Indeed, since this ST2825 chemical compound affects only the association of TIR domains of MyD88 and the disruption of this TIR domain interaction inhibits the recruitment of IRAK4. By extension, IRAK1 and the subsequent signalling cascade, dimerisation inhibition of MyD88 might affect only one specific signalling pathway [19]. These results might suggest that alternative IL-1β signalling pathways independent of MyD88 homodimerisation and recruitment in PBMC could be active as well.

This approach arises from previous studies in which new receptors associated with the recognition of IL-1β have been observed. IL-1RAcPb is a unique receptor expressed on neurons, and its expression implicates activation of certain signalling pathways, such as p38 MAPK, but not NF-κB; it has even been observed that this alternative signalling pathway is independent on MyD88, IRAK1 and TRAF6 [59,60]. Moreover, Heinz et al. reported a new molecule known as Unc5CL; this protein contains death domains (DD) similarly to those found in MyD88 [61]. Unc5CL is considered as a pro-inflammatory signalling inducer and involves recruitment of IRAK1, IRAK4, TRAF6 and converges in NF-κB and JNK activation in a MyD88-independent manner [61]. Currently, there are not reports in which these new molecules have been reported in PBMC; however, the possibility to perform future studies that elucidate this observation remains open. 

Moreover, in spite of the fact that ST2825 did not show a substantial inhibitory effect on downregulation of inflammatory cytokine from IL-1β-stimulated PBMC, it might be necessary to consider the use of other IL-1 inhibitors, such as IL-37 and IL-38 [62,63], which could be useful to compare a differential response orchestrated by ST2825 on IL-1β-stimulated PBMC and the inhibitory effect of both IL-37 and IL-38. In this regard, Conti et al. mentioned that IL-37 suppresses the innate and acquired immune response and inhibits the inflammation through its binding with IL-18 receptor-α chain (IL-18Rα) [64]. Additionally, IL-37 can activate the mTOR signalling pathway and increase the adenosine monophosphate (AMP) kinase [64]; therefore, IL-37 and IL-38 might represent cytokines of great interest in experimental models where the suppressive effect of several inflammatory mediators is required.

**Figure 4:**
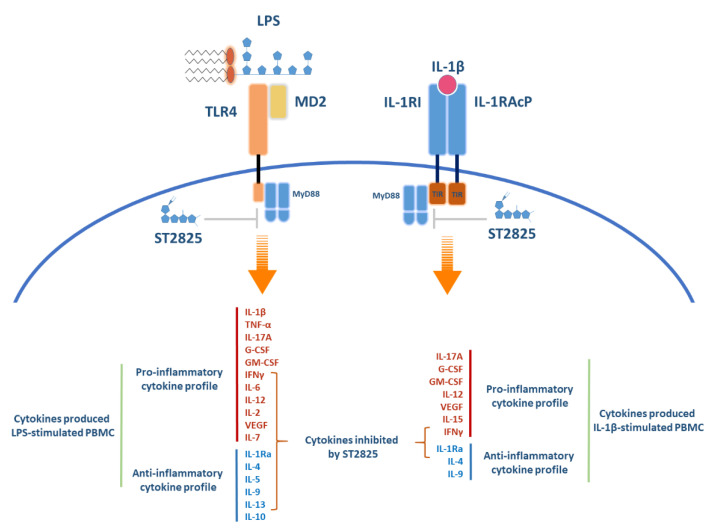
Schematic outline of cytokine release from rhIL-1β and LPS-stimulated PBMC, and cytokine inhibition orchestrated by the effect of ST2825. For illustrative purposes, we performed a representative figure to summarise our results. On the left side of this illustration, inflammatory cytokine release from LPS-stimulated PBMC is presented. On the right side of the illustration, inflammatory cytokine release from rhIL-1β-stimulated PBMC is presented. For both cases, the pro- and anti-inflammatory cytokine profiles are indicated in red and blue, respectively. The cytokines inhibited by effect of ST2825 are indicated with curly braces. LPS, lipopolysaccharide; TLR, toll-like receptor 4; MD2, myeloid differentiation factor 2; IL-1β, interleukin-1 beta; IL-1RI, IL-1 receptor type I; IL-1RAcP, interleukin 1 receptor accessory protein. TIR, toll-IL-1-receptor domains; MyD88, myeloid differentiation primary response 88. ST2825, MyD88 dimerisation inhibitor.

Furthermore, a weakness of this study was not to measure cell viability after stimulation with ST2825. Differences (that were not statistically significant) have been previously reported for ST2825 in other studies at 10, 20 or 30 μM on cell viability [19,20,58]. Our study used PBMC, reduction on specific PBMC subpopulations might contribute to the behaviour observed on these inflammatory cytokines. Therefore, considering this limitation will be important in future studies to analyse the observed response of this molecule on cell viability and the percentage of apoptosis by the effect of ST2825. The understanding of inflammatory mechanisms regarding activation and effector function on physiological processes in the first instance can provide us with an overview of the behaviour of specific factors involved in the regulation and maintenance of the inflammatory process and how it can lead to the development of chronic inflammation. This study provides information about the inhibitory activity of ST2825 on cytokine production (Figure 4), possibly through the TLR4 signalling pathway regulation in LPS-stimulated PBMC. Moreover, a relatively low impact on IL-1β signalling pathway inhibition orchestrated by ST2825 in PBMC was observed (Figure 4); possibly due to the various mechanisms of activation that remain unexplored on this signalling pathway, which could be cell subset-dependent. Nevertheless, future studies focused on the identification of specific down-stream factors implicated in both IL-1β and LPS signalling pathway activation to elucidate how the regulation of cytokine production takes place in distinct PBMC subpopulations will be required. Therefore, our results not only provide valuable evidence about the potential use of the ST2825 chemical molecule to inhibit the inflammatory cytokine release in PBMC from HBD, but also leave open the possibility to study the effect of this molecule in chronic diseases, such as rheumatic and autoimmune diseases, in which the inflammatory process plays a critical role. 

## 4. Experimental Section

### 4.1. Reagents

For PBMC stimulation LPS from *Escherichia coli* (CAT-L-2880, SIGMA^®^) (Darmstadt, Germany), Recombinant Human IL-1 beta/IL-1F2 Protein (201-LB-010, R&D Systems^®^) (Minneapolis, MN, USA), and ST2825 Inhibitor of MyD88 dimerisation (Cat. No. A3840. 894787-30-5, APExBIO) (Houston, TX, USA), were used. LPS was reconstituted in RPMI 1640 medium (GIBCO BRL, Rockville, MD, USA) and the LPS stock concentration was 10,000 ng/mL; the final concentration of LPS at 30 ng/mL was used to stimulate PBMC. ST2825 molecule was reconstituted according to the recommendations of APExBIO; final concentrations at 0, 10, 30 and 50 μM were used to determine inhibition curves for rhIL-1β and LPS. The rhIL-1β was reconstituted according to the manufacturer’s instructions. Human TNF-α concentration was determined using LEGEND MAX™ ELISA Kit with Pre-coated Plate (BioLegend, Cat. No. 430207) (San Diego, CA, USA).

### 4.2. Subjects

Ten HDB over 18 years of age were included in the study, five females and five males. A blood sample (30 mL) was collected from each participant to obtain PBMC and perform cell culture experiments. The presence of infections and body mass index ≥ 25 kg/m^2^ were taken as exclusion criteria. All subjects included in the present study signed the informed consent letter before their inclusion, and the present study was performed following the Declaration of Helsinki amendments.

### 4.3. Isolation of PBMC

The PBMC were isolated from a blood sample following the density gradient separation method using Histopaque^®^-1077 (Sigma Aldrich, St. Louis, MO, USA; ρ 1.076–1.078 g/mL). The obtained PBMC were washed and resuspended in RPMI-1640 medium supplemented with penicillin (50 U/mL, Sigma Aldrich, St. Louis, MO, USA) and streptomycin (50 μg/mL, Sigma Aldrich, St. Louis, MO, USA) at a concentration of 1% for both. The Trypan blue test analysed the cell viability, and the total of separated cells per mL was quantified directly in a Neubauer chamber.

### 4.4. Ex-Vivo Induction of Cytokines from rhIL-1β and LPS-Stimulated PBMC

The PBMC were placed in 24-well plates after the density adjustment at 1 × 10^6^ cells/mL (final volume of 1000 μL). Cells were cultured in RPMI-1640 medium without serum and supplemented with antibiotic and antifungal. Untreated cells were taken as the control group for each experiment. The effect of LPS (30 ng/mL) in the secretion of TNF-α was studied in samples from four HBD to demonstrate the positive stimulation of the cells. The concentration of ST2825 necessary to inhibit the rhIL-1β and LPS response in PBMC was determined at 0, 10, 30 and 50 μM. Subsequently, PBMC culture was performed with LPS (30 ng/mL), LPS (30 ng/mL) plus ST2825 (30 μM) or without stimulation (control group). Similarly, PBMC were stimulated with rhIL-1β (10 ng/mL), rhIL-1β (10 ng/mL) plus ST2825 (30 μM), ST2825 (30 μM) alone or without stimulation (control group). ST2825 molecule was added to the corresponding well 15 min before the stimuli with rhIL-1β or LPS. Each experiment was done with two biological replicates and was incubated for 24 h at 37 °C in a humidified 5% CO_2_ atmosphere. Once the incubation time had elapsed, supernatants were collected and stored at −80 °C for the subsequent quantification of pro-inflammatory and anti-inflammatory cytokine secretion.

### 4.5. Cytokine Analysis by Multiple Analyte Profiling (xMAP) Technology

The concentration of IL-1β, TNF-α, IFN-γ, IL-6, IL-12, IL-17A, M-CSF, GM-CSF, IL-2, VEGF, IL-15, IL-7, IL-1Ra, IL-4, IL-5, IL-13, IL-10 and IL-9 and were quantified from culture supernatants by multiplex immunoassay method using the Bio-Plex Pro^TM^ Human Cytokine 27-Plex Assay (Bio-Rad Laboratories, Inc., Hercules, CA, USA) according to the manufacturer’s instructions. Luminex MAGPIX^®^ (Luminex Corporation, Austin, TX, USA) instrument was used for xMAP assays.

### 4.6. Statistics

All data were shown as medians and interquartile ranges. The differences between the non-parametric quantitative variables were analysed by U of Mann-Whitney test to compare two groups and both the Kruskal-Wallis test and the Dunn’s post hoc test for multiple comparisons. Data analysis was performed using GraphPad Prism v5.0 software, and a *p*-value < 0.05 was considered statistically significant.

## Figures and Tables

**Table 1:** Effect of ST2825 on rhIL-1β-stimulated PBMC.

	**Pro-Inflammatory Cytokine Profile**				
	**RPMI**	**rhIL-1β**	* *p*-Value	**rhIL-1β + ST2825**	** *p*-Value
TNF-α	825.3 (304.3–2020)	1158 (648–1918)	ns	409 (289.5–303.8)	ns
IFN-γ	70.3 (18.6–126.6)	167.8 (137.4–184)	ns	26.52 (24.20–33.3)	<0.01
IL-6	434 (171.4–1333)	894.2 (720.4–1795)	ns	982.3 (713.3–2371)	ns
IL-12	1.4 (0.4–1.4)	4.8 (4.4–5.1)	<0.01	4.7 (3.2–5.1)	ns
IL-17A	12.2 (6.3–23.7)	86.8 (74–88.4)	<0.05	81.1 (71–86.2)	ns
G-CSF	131.8 (82.1–243.6)	293.6 (241.9–534.8)	<0.05	402.2 (277.1–573.2)	ns
GM-CSF	1.4 (0.8–2.6)	5.4 (4.9–5.8)	<0.05	5.2 (4–5.7)	ns
IL-2	18.1 (8.1–38.2)	41.1 (37.3–49.6)	ns	28.5 (24.8–29.9)	ns
VEGF	0 (0–88.5)	723.4 (586.2–805.3)	<0.05	843.2 (585.1–954.2)	ns
IL-15	1.2 (0–17.4)	74.1 (69.7–77.3)	<0.05	78.06 (70.65–79.3)	ns
IL-7	14.2 (7.7–17.7)	28.7 (22.1–30.7)	ns	25.7 (16.9–30.1)	ns
	**Anti-Inflammatory Cytokine Profile**				
	**RPMI**	**rhIL-1β**	* *p*-Value	**rhIL-1β + ST2825**	** *p*-Value
IL-1Ra	2211 (1165–3543)	3339 (2399–3710)	ns	670 (426–1638)	<0.001
IL-4	3 (1.6–5.6)	13.7 (12.8–14.6)	<0.01	13.5 (12.3–14)	ns
IL-5	17.9 (8.6–40.5)	53 (49.1–60)	ns	45.8 (37.4–57.3)	ns
IL-13	0.6 (0.2–0.8)	1.1 (0.9–1.2)	ns	1 (0.9–1.1)	ns
IL-10	13.3 (5.6–50.5)	43 (27.1–83.6)	ns	22.8 (13.2–35.6)	ns
IL-9	7.9 (5.3–10.5)	14 (12.3–15.3)	<0.01	12.7 (10–16.3)	ns

Data provided in medians and interquartile ranges (25th and 75th). The concentration of cytokines is provided in pg/mL. * *p*-value determined by Dunn’s test (RPMI vs rhIL-1β). ** *p*-value determined by Dunn’s test (rhIL-1β vs. rhIL-1β plus ST2825). Not significant (ns). Abbreviations: IL, interleukin; TNF-α, tumor necrosis factor alpha; IFN-γ, interferon gamma; G-CSF, granulocyte colony-stimulating factor; GM-CSF, granulocyte-monocyte colony-stimulating factor; VEGF, vascular endothelial growth factor.

## Supplementary Materials

The following are available online, Table S1: Effect of ST2825 on LPS-stimulated PBMC, Table S2: Effect of ST2825 alone on inflammatory cytokine production.

## References

[1] Deguine J., Barton G.M. (2014). MyD88: A central player in innate immune signaling. F1000Prime Rep..

[2] Balka K.R., De Nardo D. (2018). Understanding early TLR signaling through the Myddosome. J. Leukoc. Biol..

[3] Dinarello C.A. (2019). The IL-1 family of cytokines and receptors in rheumatic diseases. Nat. Rev. Rheumatol..

[4] Newton K., Dixit V.M. (2012). Signaling in innate immunity and inflammation. Cold Spring Harb. Perspect. Biol..

[5] Elshabrawy H.A., Essani A.E., Szekanecz Z., Fox D.A., Shahrara S. (2017). TLRs, future potential therapeutic targets for RA. Autoimmun. Rev..

[6] Boraschi D., Tagliabue A. (2013). The interleukin-1 receptor family. Semin. Immunol..

[7] Akira S., Takeda K., Kaisho T. (2001). Toll-like receptors: Critical proteins linking innate and acquired immunity. Nat. Immunol..

[8] Vijay K. (2018). Toll-like receptors in immunity and inflammatory diseases: Past, present, and future. Int. Immunopharmacol..

[9] Rossol M., Heine H., Meusch U., Quandt D., Klein C., Sweet M.J., Hauschildt S. (2011). LPS-induced cytokine production in human monocytes and macrophages. Crit. Rev. Immunol..

[10] Brinson C.W., Lu Z., Li Y., Lopes-Virella M.F., Huang Y. (2016). Lipopolysaccharide and IL-1β coordinate a synergy on cytokine production by upregulating MyD88 expression in human gingival fibroblasts. Mol. Immunol..

[11] Lappas M. (2017). The IL-1β signalling pathway and its role in regulating pro-inflammatory and pro-labour mediators in human primary myometrial cells. Reprod. Biol..

[12] Scuderi F., Convertino R., Molino N., Provenzano C., Marino M., Zoli A., Bartoccioni E. (2003). Effect of pro-inflammatory/anti-inflammatory agents on cytokine secretion by peripheral blood mononuclear cells in rheumatoid arthritis and systemic lupus erythematosus. Autoimmunity.

[13] Smedman C., Gårdlund B., Nihlmark K., Gille-Johnson P., Andersson J., Paulie S. (2009). ELISpot analysis of LPS-stimulated leukocytes: Human granulocytes selectively secrete IL-8, MIP-1beta and TNF-alpha. J. Immunol. Methods.

[14] Chovanova L., Vlcek M., Krskova K., Penesova A., Radikova Z., Rovensky J., Cholujova D., Sedlak J., Imrich R. (2013). Increased production of IL-6 and IL-17 in lipopolysaccharide-stimulated peripheral mononuclears from patients with rheumatoid arthritis. Gen Physiol. Biophys..

[15] Van Dooren F.H., Duijvis N.W., te Velde A.A. (2013). Analysis of cytokines and chemokines produced by whole blood, peripheral mononuclear and polymorphonuclear cells. J. Immunol. Methods.

[16] Olson M.A., Lee M.S., Kissner T.L., Alam S., Waugh D.S., Saikh K.U. (2015). Discovery of small molecule inhibitors of MyD88-dependent signaling pathways using a computational screen. Sci. Rep..

[17] Avbelj M., Horvat S., Jerala R. (2011). The role of intermediary domain of MyD88 in cell activation and therapeutic Inhibition of TLRs. J. Immunol..

[18] Li J., Wang X., Zhang F., Yin H. (2013). Toll-like receptors as therapeutic targets for autoimmune connective tissue diseases. Pharmacol. Ther..

[19] Loiarro M., Capolunghi F., Fantò N., Gallo G., Campo S., Arseni B., Carsetti R., Carminati P., De Santis R., Ruggiero V. (2007). Pivotal Advance: Inhibition of MyD88 dimerisation and recruitment of IRAK1 and IRAK4 by a novel peptidomimetic compound. J. Leukoc. Biol..

[20] Capolunghi F., Rosado M.M., Cascioli S., Girolami E., Bordasco S., Vivarelli M., Ruggiero B., Cortis E., Insalaco A., Fantò N. (2010). Pharmacological inhibition of TLR9 activation blocks autoantibody production in human B cells from SLE patients. Rheumatology.

[21] Loiarro M., Ruggiero V., Sette C. (2013). Targeting the Toll-like receptor/interleukin 1 receptor pathway in human diseases: Rational design of MyD88 inhibitors. Clin. Lymphoma Myeloma Leuk..

[22] Long T., Liu Z., Shang J., Zhou X., Yu S., Tian H., Bao Y. (2018). Polygonatum sibiricum polysaccharides play anti-cancer effect through TLR4-MAPK/NF-κB signaling pathways. Int. J. Biol. Macromol..

[23] Weber A., Wasiliew P., Kracht M. (2010). Interleukin-1 (IL-1) pathway. Sci. Signal..

[24] Hua H., Du Y., Ma R., Zhang B.-B., Yu Q., Li B., Xu J.-T., Li X.-Y., Tang R.-X., Yan C. (2018). The Regulatory Roles of Toll-Like Receptor 4 in Secretions of Type 1/Type 2 Relative Cytokines by Splenocytes and Dendritic Cells Exposed to Clonorchis sinensis Excretory/Secretory Products. Inflammation.

[25] Shirakawa F., Tanaka Y., Ota T., Suzuki H., Eto S., Yamashita U. (1987). Expression of interleukin 1 receptors on human peripheral T cells. J. Immunol..

[26] Garlanda C., Dinarello C.A., Mantovani A. (2013). The interleukin-1 family: Back to the future. Immunity.

[27] Vasilyev F.F., Lopatnikova J.A., Sennikov S.V. (2013). Optimised flow cytometry protocol for analysis of surface expression of interleukin-1 receptor types I and II. Cytotechnology.

[28] Shiratori H., Feinweber C., Luckhardt S., Linke B., Resch E., Geisslinger G., Weigert A., Parnham M.J. (2017). THP-1 and human peripheral blood mononuclear cell-derived macrophages differ in their capacity to polarise in vitro. Mol. Immunol..

[29] Bhattacharya S., Aggarwal A. (2018). M2 macrophages and their role in rheumatic diseases. Rheumatol. Int..

[30] Lechner J., Chen M., Hogg R.E., Toth L., Silvestri G., Chakravarthy U., Xu H. (2017). Peripheral blood mononuclear cells from neovascular age-related macular degeneration patients produce higher levels of chemokines CCL2 (MCP-1) and CXCL8 (IL-8). J. Neuroinflamm..

[31] Rothfuchs A.G., Trumstedt C., Wigzell H., Rottenberg M.E. (2004). Intracellular bacterial infection-induced IFN-gamma is critically but not solely dependent on Toll-like receptor 4-myeloid differentiation factor 88-IFN-alpha beta-STAT1 signaling. J. Immunol..

[32] Park J.-H., Jeong S.-Y., Choi A.-J., Kim S.-J. (2015). Lipopolysaccharide directly stimulates Th17 differentiation in vitro modulating phosphorylation of RelB and NF-κB1. Immunol. Lett..

[33] Peyssonnaux C., Cejudo-Martin P., Doedens A., Zinkernagel A.S., Johnson R.S., Nizet V. (2007). Cutting edge: Essential role of hypoxia inducible factor-1alpha in development of lipopolysaccharide-induced sepsis. J. Immunol..

[34] Matsui-Hasumi A., Sato Y., Uto-Konomi A., Yamashita S., Uehori J., Yoshimura A., Yamashita M., Asahara H., Suzuki S., Kubo M. (2017). E3 ubiquitin ligases SIAH1/2 regulate hypoxia-inducible factor-1 (HIF-1)-mediated Th17 cell differentiation. Int. Immunol..

[35] Nyati K.K., Masuda K., Zaman M.M.-U., Dubey P.K., Millrine D., Chalise J.P., Higa M., Li S., Standley D.M., Saito K. (2017). TLR4-induced NF-κB and MAPK signaling regulate the IL-6 mRNA stabilising protein Arid5a. Nucleic Acids Res..

[36] El Chartouni C., Schwarzfischer L., Rehli M. (2010). Interleukin-4 induced interferon regulatory factor (Irf) 4 participates in the regulation of alternative macrophage priming. Immunobiology.

[37] Negishi H., Ohba Y., Yanai H., Takaoka A., Honma K., Yui K., Matsuyama T., Taniguchi T., Honda K. (2005). Negative regulation of Toll-like-receptor signaling by IRF-4. Proc. Natl. Acad. Sci. USA.

[38] Pena O.M., Pistolic J., Raj D., Fjell C.D., Hancock R.E.W. (2011). Endotoxin tolerance represents a distinctive state of alternative polarisation (M2) in human mononuclear cells. J. Immunol..

[39] O’Carroll C., Fagan A., Shanahan F., Carmody R.J. (2014). Identification of a unique hybrid macrophage-polarisation state following recovery from lipopolysaccharide tolerance. J. Immunol..

[40] Sutton C.E., Lalor S.J., Sweeney C.M., Brereton C.F., Lavelle E.C., Mills K.H.G. (2009). Interleukin-1 and IL-23 induce innate IL-17 production from gammadelta T cells, amplifying Th17 responses and autoimmunity. Immunity.

[41] Cai Y., Shen X., Ding C., Qi C., Li K., Li X., Jala V.R., Zhang H., Wang T., Zheng J. (2011). Pivotal role of dermal IL-17-producing γδ T cells in skin inflammation. Immunity.

[42] Chung Y., Chang S.H., Martinez G.J., Yang X.O., Nurieva R., Kang H.S., Ma L., Watowich S.S., Jetten A.M., Tian Q. (2009). Critical regulation of early Th17 cell differentiation by interleukin-1 signaling. Immunity.

[43] Hu Y., Xu F., Zhang R., Legarda D., Dai J., Wang D., Li H., Zhang Y., Xue Q., Dong G. (2019). Interleukin-1β-induced IRAK1 ubiquitination is required for TH-GM-CSF cell differentiation in T cell-mediated inflammation. J. Autoimmun..

[44] Langlet C., Springael C., Johnson J., Thomas S., Flamand V., Leitges M., Goldman M., Aksoy E., Willems F. (2010). PKC-alpha controls MYD88-dependent TLR/IL-1R signaling and cytokine production in mouse and human dendritic cells. Eur. J. Immunol..

[45] Thornton R.D., Lane P., Borghaei R.C., Pease E.A., Caro J., Mochan E. (2000). Interleukin 1 induces hypoxia-inducible factor 1 in human gingival and synovial fibroblasts. Biochem. J..

[46] Jung Y.-J., Isaacs J.S., Lee S., Trepel J., Neckers L. (2003). IL-1beta-mediated up-regulation of HIF-1alpha via an NFkappaB/COX-2 pathway identifies HIF-1 as a critical link between inflammation and oncogenesis. FASEB J..

[47] Sartori-Cintra A.R., de Mara C.S., Argolo D.L., Coimbra I.B. (2012). Regulation of hypoxia-inducible factor-1α (HIF-1α) expression by interleukin-1β (IL-1 β), insulin-like growth factors I (IGF-I) and II (IGF-II) in human osteoarthritic chondrocytes. Clinics.

[48] Yao H., Hu C., Yin L., Tao X., Xu L., Qi Y., Han X., Xu Y., Zhao Y., Wang C. (2016). Dioscin reduces lipopolysaccharide-induced inflammatory liver injury via regulating TLR4/MyD88 signal pathway. Int. Immunopharmacol..

[49] Yang X., Chen G.T., Wang Y.Q., Xian S., Zhang L., Zhu S.M., Pan F., Cheng Y.X. (2018). TLR4 promotes the expression of HIF-1α by triggering reactive oxygen species in cervical cancer cells in vitro-implications for therapeutic intervention. Mol. Med. Rep..

[50] Deng Y., Sun J., Zhang L.D. (2016). Effect of ST2825 on the proliferation and apoptosis of human hepatocellular carcinoma cells. Genet. Mol. Res..

[51] Zhang H.-S., Li H., Zhang D.-D., Yan H.-Y., Zhang Z.-H., Zhou C.-H., Ye Z.-N., Chen Q., Jiang T.-W., Liu J.-P. (2016). Inhibition of myeloid differentiation factor 88(MyD88) by ST2825 provides neuroprotection after experimental traumatic brain injury in mice. Brain Res..

[52] Shiratori E., Itoh M., Tohda S. (2017). MYD88 Inhibitor ST2825 Suppresses the Growth of Lymphoma and Leukaemia Cells. Anticancer Res..

[53] Dinarello C.A. (2013). Overview of the interleukin-1 family of ligands and receptors. Semin. Immunol..

[54] Palomo J., Dietrich D., Martin P., Palmer G., Gabay C. (2015). The interleukin (IL)-1 cytokine family—Balance between agonists and antagonists in inflammatory diseases. Cytokine.

[55] Li C., Zienkiewicz J., Hawiger J. (2005). Interactive sites in the MyD88 Toll/interleukin (IL) 1 receptor domain responsible for coupling to the IL1beta signaling pathway. J. Biol. Chem..

[56] Loiarro M., Sette C., Gallo G., Ciacci A., Fantò N., Mastroianni D., Carminati P., Ruggiero V. (2005). Peptide-mediated interference of TIR domain dimerisation in MyD88 inhibits interleukin-1-dependent activation of NF-{kappa}B. J. Biol. Chem..

[57] Ahmad R., Sylvester J., Zafarullah M. (2007). MyD88, IRAK1 and TRAF6 knockdown in human chondrocytes inhibits interleukin-1-induced matrix metalloproteinase-13 gene expression and promoter activity by impairing MAP kinase activation. Cell. Signal..

[58] Wang X., Tan Y., Huang Z., Huang N., Gao M., Zhou F., Hu J., & Feng W. (2019). Disrupting myddosome assembly in diffuse large B-cell lymphoma cells using the MYD88 dimerisation inhibitor ST2825. Oncol. Rep..

[59] Huang Y., Smith D.E., Ibáñez-Sandoval O., Sims J.E., Friedman W.J. (2011). Neuron-specific effects of interleukin-1β are mediated by a novel isoform of the IL-1 receptor accessory protein. J. Neurosci..

[60] Smith D.E., Lipsky B.P., Russell C., Ketchem R.R., Kirchner J., Hensley K., Huang Y., Friedman W.J., Boissonneault V., Plante M.-M. (2009). A central nervous system-restricted isoform of the interleukin-1 receptor accessory protein modulates neuronal responses to interleukin-1. Immunity.

[61] Heinz L.X., Rebsamen M., Rossi D.C., Staehli F., Schroder K., Quadroni M., Gross O., Schneider P., Tschopp J. (2012). The death domain-containing protein Unc5CL is a novel MyD88-independent activator of the pro-inflammatory IRAK signaling cascade. Cell Death Differ..

[62] E Gallenga C., Pandolfi F., Caraffa A., Kritas S.K., Ronconi G., Toniato E., Martinotti S., Conti P. (2019). Interleukin-1 family cytokines and mast cells: Activation and inhibition. J. Biol. Regul. Homeost. Agents.

[63] Caraffa A., Gallenga C.E., Kritas S.K., Ronconi G., Di Emidio P., Conti P. (2019). CAR-T cell therapy causes inflammation by IL-1 which activates inflammatory cytokine mast cells: Anti-inflammatory role of IL-37. J. Biol. Regul. Homeost. Agents.

[64] Conti P., Ronconi G., Caraffa A., Gallenga C.E., Ross R., Frydas I., Kritas S.K. (2020). Induction of pro-inflammatory cytokines (IL-1 and IL-6) and lung inflammation by Coronavirus-19 (COVI-19 or SARS-CoV-2): Anti-inflammatory strategies. J. Biol. Regul. Homeost. Agents.

