# Supplementary material for: Downregulation of Inflammatory Cytokine Release from IL-1β and LPS-Stimulated PBMC Orchestrated by ST2825, a MyD88 Dimerisation Inhibitor
Source: Molecules. 2020 Sep 21;25(18):4322. doi: 10.3390/molecules25184322 (PMC7570868; doi:10.3390/molecules25184322)
Supplement: Supplementary file 1 [file molecules-25-04322-s001.pdf]

Table S1. Effect of ST2825 on LPS-stimulated PBMC. PBMC were stimulated with LPS (30 ng/mL) in presence or absence of ST2825 (30 μM).

	Pro-inflammatory cytokine profile				
	RPMI	LPS	**p*-value	LPS + ST2825	***p*-value
**IL-1β**	26 (6-82)	3069 (2824-3340)	<0.0001	1569 (1244-2511)	ns
**TNF-α**	825 (304-2020)	18146 (12060-23087)	<0.001	3705 (3270-5820)	ns
**IFN-γ**	70 (19-127)	235 (173-383)	<0.01	27 (20-39)	<0.001
**IL-6**	434 (171-1333)	3489 (2985-6492)	<0.001	2036 (1303-2604)	<0.05
**IL-12**	1.4 (0.4-1.4)	18.1 (15.5-24.4)	<0.001	6.1 (4.7-6.3)	<0.05
**IL-17A**	12.2 (6.3-23.7)	231 (196.4-277.2)	<0.001	82 (62.4-85.9)	ns
**G-CSF**	131.8 (82.1-243.6)	3043 (2020-4270)	<0.001	1360 (1086-2758)	ns
**GM-CSF**	1.4 (0.8-2.6)	21.5 (18.8-33.3)	<0.001	10 (6.5-13.9)	ns
**IL-2**	18.1 (8.1-38.2)	296.3 (175.3-331.7)	<0.001	73 (62.4-96.1)	<0.05
**VEGF**	0 (0-88.53)	2153 (1913-2551)	<0.001	540.1 (352.4-952)	<0.05
**IL-15**	1.2 (0-17.4)	214 (190.5-275.8)	<0.001	73.8 (57-83.3)	<0.05
**IL-7**	14.2 (7.7-17.7)	68.6 (39.5-96.4)	<0.001	18.3 (4.3-21)	<0.01
	Anti-inflammatory cytokine profile				
	RPMI	LPS	**p*-value	LPS + ST2825	***p*-value
**IL-1Ra**	2211 (1165-3543)	9035 (7213-12693)	<0.01	1900 (1442-3324)	<0.001
**IL-4**	3 (1.6-5.6)	36.4 (27.9-50.6)	<0.001	14.1 (12.3-16.1)	<0.05
**IL-5**	17.9 (8.6-40.5)	172.4 (134.5-208.5)	<0.001	45.8 (39.1-51.8)	<0.05
**IL-13**	0.6 (0.2-0.8)	3.4 (2.7-4.9)	<0.001	0.6 (0.5-0.9)	<0.01
**IL-10**	13.3 (5.6-50.5)	1162 (759.8-2056)	<0.001	354.7 (293.2-452.8)	ns
**IL-9**	7.9 (5.3-10.5)	42.7 (39.4-51)	<0.001	12.3 (10.2-14.6)	<0.05

Data provided in medians and interquartile ranges (25th and 75th). The concentration of cytokines is provided in pg/mL. * *p*-value determined by Dunn's test (RPMI vs LPS). ** *p*-value determined by Dunn's test (LPS vs LPS plus ST2825).

Table S2. Effect of ST2825 alone on inflammatory cytokine production. PBMC in presence or absence of ST2825 (30 μM).

	Pro-inflammatory cytokine profile		
	RPMI	ST2825	**p*-value
**IL-1β**	26 (6-82)	27.81 (5-84)	ns
**TNF-α**	825 (304-2020)	101 (25-197)	<0.01
**IFN-γ**	70 (19-127)	2 (0-11)	<0.01
**IL-6**	434 (171-1333)	214 (28-853)	ns
**IL-12**	1.4 (0.4-1.4)	1.8 (0.7-2.3)	ns
**IL-17A**	12.2 (6.3-23.7)	7.7 (2.1-15.8)	ns
**G-CSF**	131.8 (82.1-243.6)	114.8 (30.7-221.5)	ns
**GM-CSF**	1.4 (0.8-2.6)	1 (0.02-1.7)	ns
**IL-2**	18.1 (8.1-38.2)	5.6 (1.6-10.9)	<0.05
**VEGF**	0 (0-88.53)	0 (0-0)	ns
**IL-15**	1.2 (0-17.4)	0 (0-15.2)	ns
**IL-7**	14.2 (7.7-17.7)	14.8 (7.7-19.3)	ns

	Anti-inflammatory cytokine profile		
	RPMI	ST2825	**p*-value
**IL-1Ra**	2211 (1165-3543)	304 (124-855)	<0.01
**IL-4**	3 (1.6-5.6)	2.2 (0.9-3.5)	ns
**IL-5**	17.9 (8.6-40.5)	12.4 (2.7-23.5)	ns
**IL-13**	0.6 (0.2-0.8)	0.6 (0.2-0.7)	ns
**IL-10**	13.3 (5.6-50.5)	6.6 (0.3-14.1)	ns
**IL-9**	7.9 (5.3-10.5)	5.6 (4.4-10.2)	ns

Data provided in medians and interquartile ranges (25th and 75th). The concentration of cytokines is provided in pg/mL. * *p*-value determined by Mann-Whitney U test (RPMI vs ST2825).
